# Naturally colored cotton for wearable applications

**DOI:** 10.3389/fpls.2024.1350405

**Published:** 2024-03-21

**Authors:** Marina Naoumkina, Doug J. Hinchliffe, Gregory N. Thyssen

**Affiliations:** Cotton Fiber Bioscience and Utilization Research Unit, United States Department of Agriculture (USDA), Agricultural Research Service (ARS), Southern Regional Research Center (SRRC), New Orleans, LA, United States

**Keywords:** naturally colored cotton, proanthocyanidin pigments, flame retardance, condensed tannin, flavan-3-ols

## Abstract

Naturally colored cotton (NCC) offers an environmentally friendly fiber for textile applications. Processing white cotton fiber into textiles requires extensive energy, water, and chemicals, whereas processing of NCC skips the most polluting activity, scouring-bleaching and dyeing; therefore, NCC provides an avenue to minimize the harmful impacts of textile production. NCC varieties are suitable for organic agriculture since they are naturally insect and disease-resistant, salt and drought-tolerant. Various fiber shades, ranging from light green to tan and brown, are available in the cultivated NCC (*Gossypium hirsutum* L.) species. The pigments responsible for the color of brown cotton fiber are proanthocyanidins or their derivatives synthesized by the flavonoid pathway. Due to pigments, the NCC has excellent ultraviolet protection properties. Some brown cotton varieties exhibited superior thermal resistance of fiber that can be used to make fabrics with enhanced flame retardancy. Here, we review molecular mechanisms involved in the pigment production of brown cotton and challenges in breeding NCC varieties with a wide range of colors but without penalty in fiber quality. Also, we discuss opportunities for NCC with flame-retarding properties in textile applications.

## Introduction

**Figure 1:**
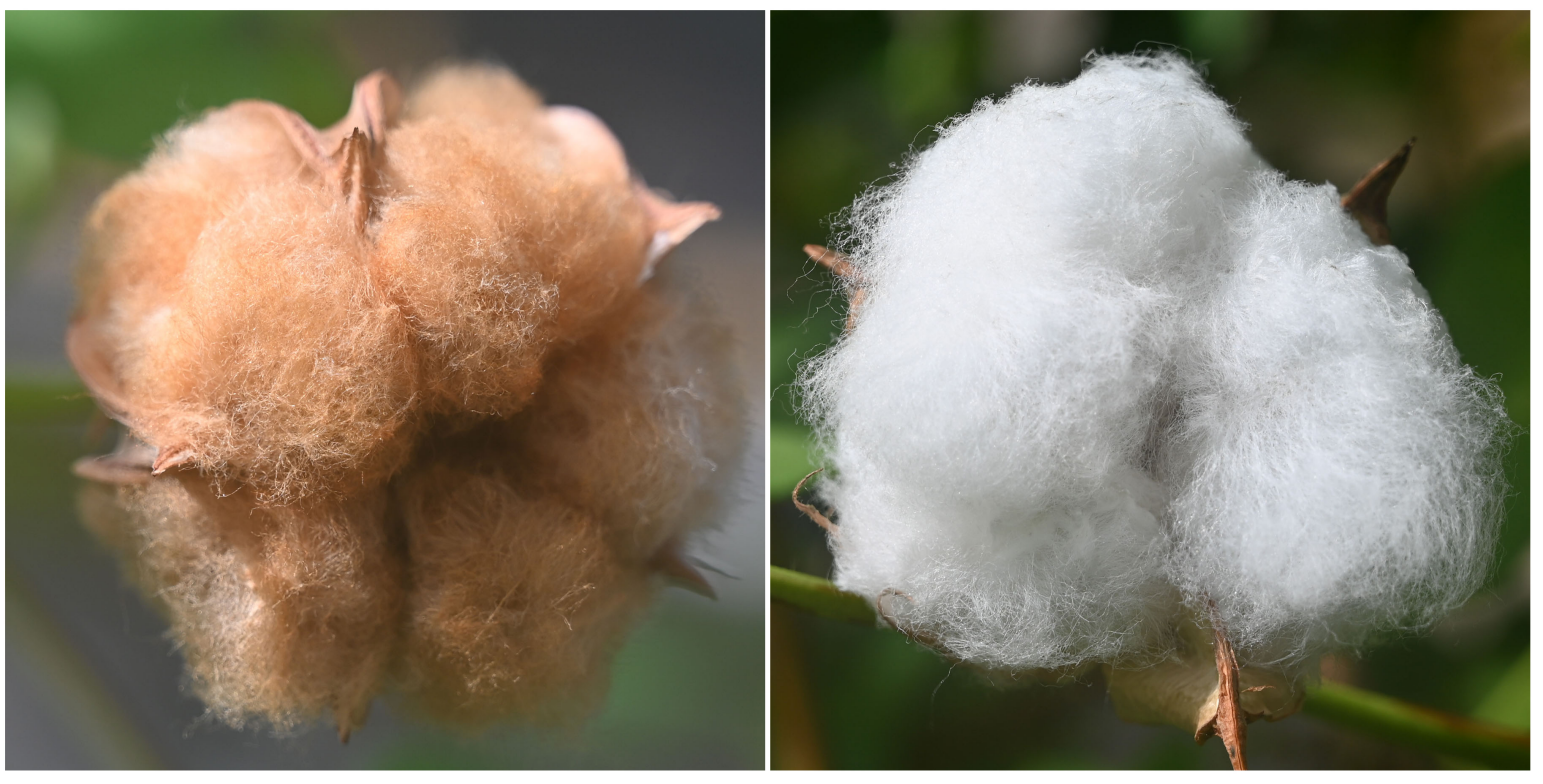
Open bolls of brown and white cotton.

Naturally colored cotton (NCC) fibers exist in different hues of brown, red, rust, and green and can be used as eco-friendly alternatives to white cotton (**Figure 1**). The conventional process of dyeing white cotton requires extensive water, energy, and chemicals and contributes about 15% to the cost of the finished garment (Nimon and Beghin, 1999). NCC fabrics resist fading since colors become stronger after laundering (Günaydin et al., 2019). Due to pigments, the clothes made from NCC fibers protect the skin from ultraviolet radiation. Fibers of some brown cotton varieties exhibited flame retardant (FR) properties, making them suitable for specific end-use applications, such as automotive interiors (Parmar and Chakraborty, 2001; Hinchliffe et al., 2015, 2016; Nam et al., 2016, 2017).

Despite these advantages, typical NCC fibers are weaker and shorter than cultivated white cotton fibers, deeming them unsuitable for high-speed woven textile machinery. The breeding efforts in the 1990s resulted in the release of improved brown fiber germplasm lines (May et al., 1994). However, the major obstacle to expanding NCC production is not marketing but regulations to protect white cotton from contamination during ginning and the cost associated with cleaning equipment (Funk and Gamble, 2009). Limited color diversity is another hindrance to expanding the NCC to commercial textile production (Dutt et al., 2004; Yuan et al., 2013). Currently, NCC occupies a textile market niche promoting environmentally friendly clothing production.

Nevertheless, the use of NCC has great merit and should be expanded since global textile production generates toxic chemical waste with a negative impact on the environment. A recent forum article reviewed molecular mechanisms underlying pigmentation in brown and green cotton and suggested “omics-driven breeding” for better fiber quality NCC (Sun et al., 2021). Here, we review the regulation of pigment development in brown cotton and possible biotechnological strategies to increase hue diversity and improve fiber yield and quality. Also, we discuss the source of FR in brown cotton.

## Pigments in brown cotton fibers

**Figure 2:**
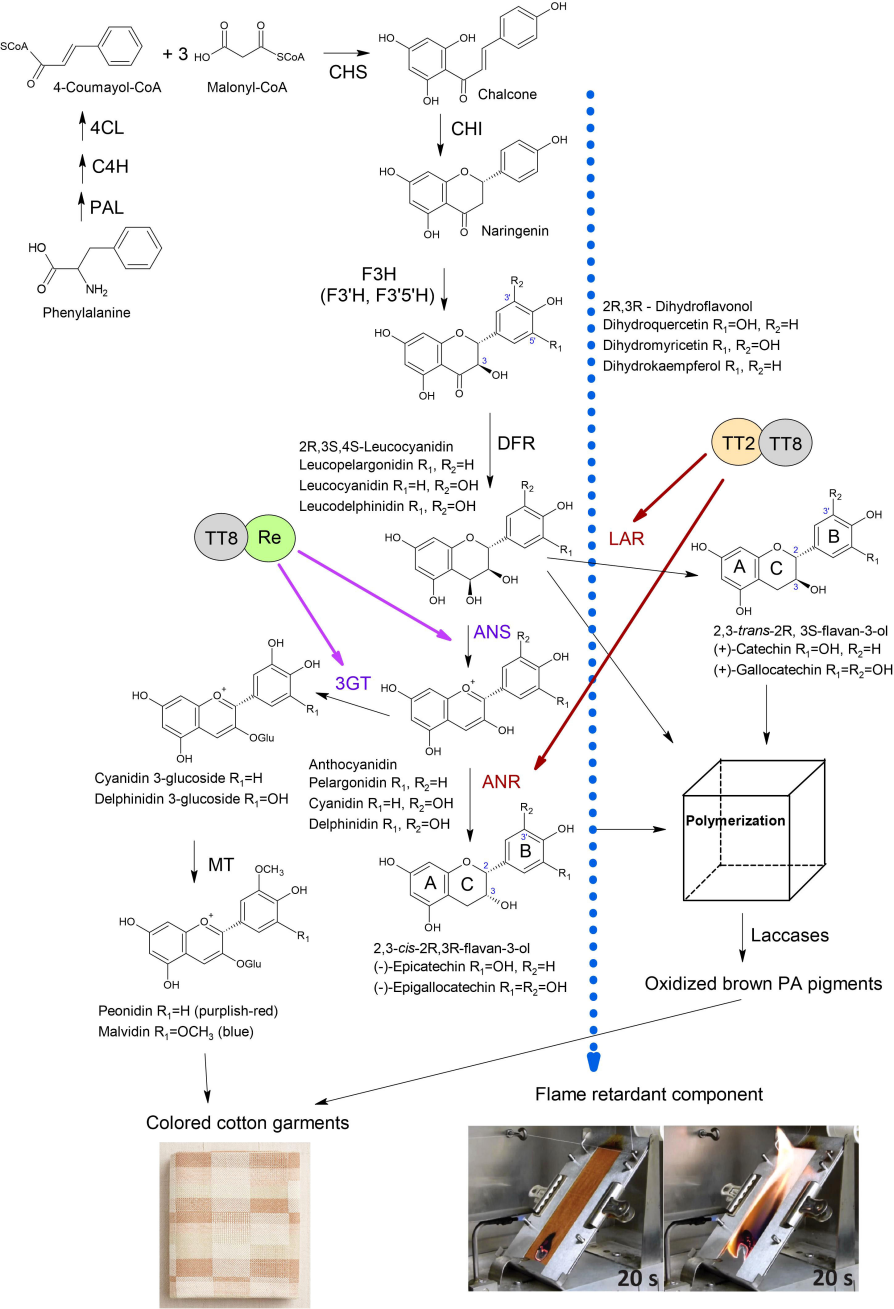
Anthocyanidin and proanthocyanidin biosynthesis in brown cotton. PAL, phenylalanine ammonia-lyase; C4H, cinnamate 4-hydroxylase; 4CL, 4-coumarate CoA ligase; CHS, chalcone synthase; CHI, chalcone isomerase; F3H, flavanone 3-hydroxylase; F3’H, flavonoid 3’-hydroxylase; F3’5’H, flavonoid 3’,5’-hydroxylase; DFR, dihydroflavonol 4-reductase; LAR, leucoanthocyanidin reductase; ANS, anthocyanidin synthase; 3GT, 3-O-glucosyltransferase; ANR, anthocyanidin reductase; MT, methyltransferase. The Blue dotted arrow indicates the transport of an unknown FR compound. Images of the flammability test of brown and white cotton fabrics were adopted from the report (Hinchliffe et al., 2016). Brown arrows indicate the activation of LAR and ANR by the TT2-TT8 complex reported by (Yan et al., 2018). Purple arrows indicate activation of ANS and 3GT by Re-TT8 complex reported by (Wang et al., 2022).

Brown cotton varieties are most commonly used for NCC fabrics. Proanthocyanidins (PAs) are the primary pigments responsible for brown fibers. PAs, also called condensed tannins, are polymeric flavan-3-ols with the diphenylpropane typical chemical structure (C6-C3-C6), which includes a benzopyran (A and C rings) linked with another aromatic ring (B ring) at C2 position (**Figure 2**) (Yu et al., 2023). The adjacent subunits of PA are linked by C4-C8 or C4-C6 carbon-carbon bonds (Xie et al., 2004). PAs were initially detected in brown fibers with DMACA (*p*-Dimethylaminocinnamaldehyde) staining. A comparative study of the treatment of mature white, green, and brown fibers with DMACA determined that only brown fibers turned blue, while white and green fibers did not change color (Xiao et al., 2007). However, PAs in minimal quantities were detected in developing white fibers from 5 to 15 days post-anthesis (DPA) and, after that, gradually disappeared (Li et al., 2012). In contrast, PAs were detected as early as 3 DPA in developing brown fibers and were synthesized in high quantities up to maturation (Li et al., 2012).

The common flavan-3-ols are (+)-catechin (2,3-*trans*) and (-)-epicatechin (2,3-*cis*) with the gallate modification of the hydroxyl group at the C3’ position on the B-ring (**Figure 2**) (Dixon et al., 2005; Xie and Dixon, 2005). The structure variability of PAs depends on the 2,3- stereochemistry of the starter and extension units and additional derivatizations such as O-methylation, O-acylation, C- and O-glycosylation (Dixon and Sarnala, 2020). The structure of PA units in brown fibers was studied with nuclear magnetic resonance (NMR), a matrix-assisted laser desorption/ionization-time of flight mass spectrometry (MALDI-TOF MS), and liquid-chromatography mass spectrometry (LC-MS) (Feng et al., 2014; Xiao et al., 2014). In one study, LC-MS analyses determined that the most abundant flavan-3-ols in brown fiber were in 2,3-*trans* form (catechin and gallocatechin), and leucoanthocyanidin reductase (LAR) was highly expressed (Xiao et al., 2014). Another work showed by NMR and MALDI-TOF MS analyses that the most predominant PA units were 2,3-*cis* form (epicatechin and epigallocatechin), and anthocyanidin reductase (ANR) was a key gene in brown fiber pigment biosynthesis (Feng et al., 2014). Therefore, both flavan-3-ols forms, 2,3-*trans* and 2,3-*cis* units, can be produced in brown cotton. The dimeric PAs, procyanidin (PC) and prodelphidin (PD) were detected in white and brown fibers; however, acylation modified some PAs in white fibers (Feng et al., 2014). The ratio of PC and PD was equal in white fibers, whereas brown cotton fibers contained mainly PD units with a relative ratio of 9:1. Feng et al. (2014) suggested that quinones, the oxidation products of proanthocyanidins, directly contribute to color development in brown fiber because developing fibers do not show distinct coloration until maturation.

## PA biosynthesis in brown cotton

PAs are synthesized through the flavonoid pathway, a branch of the phenylpropanoid pathway (Yu et al., 2023). Phenylalanine is converted into 4-Coumaroyl-CoA through a few enzymatic steps by phenylalanine ammonia-lyase (PAL), cinnamate 4-hydroxylase (C4H), and 4-coumarate CoA ligase (4CL) (**Figure 2**). Chalcone synthase (CHS) then catalyzes chalcone from one molecule of 4-coumaroyl-CoA and three molecules of malonyl-CoA. Chalcone isomerase (CHI) catalyzes the cyclization of chalcone into flavanone naringenin. Flavanone 3/3’/3’5’hydroxylases (F3H/F3’H/F3’5’H) add hydroxyl groups to the C-3, C-3’, and C-5’ of the naringenin, generating dihydrokaempferol, dihydroquercetin, or dihydromyricetin. Dihydroflavonol 4-reductase (DFR) reduces the carbonyl group at the C4 position to a hydroxyl group, producing leucoanthocyanidins (**Figure 2**). Leucoanthocyanidins are converted into flavan-3-ols in two ways: 1) LAR catalases the reductive removal of the hydroxyl at the C4 position producing 2,3-*trans* isoforms; 2) anthocyanidin synthase (ANS) catalases production of anthocyanidins, which are converted by ANR into 2,3-*cis* flavan-3-ol monomers (Xie et al., 2003). It has been demonstrated by *in vitro* enzyme assays and virus-induced gene silencing (VIGS) that the ANR pathway contributes to the biosynthesis of 2,3-*cis* flavan-3-ol in cotton (Zhu et al., 2015).

Transcriptomic and proteomic studies in cotton identified that a set of structural genes of the flavonoid pathway, including *PAL*, *C4H*, *4CL*, *CHS*, *CHI*, *F3H, F3’H*, *DFR*, *LAR*, *ANS*, *ANR*, were up-regulated in brown fibers (Li et al., 2013; Feng et al., 2014; Xiao et al., 2014; Hinchliffe et al., 2016; Gao et al., 2019; Peng et al., 2020; Canavar and Rausher, 2021). Several structural genes of the flavonoid pathway were characterized in brown cotton. VIGS of *GhCHS2*, *GhLAR1*, and *GhANR* significantly reduced intensity of the color of brown fibers and anthocyanidin content (Gao et al., 2019). RNAi silencing of *GhCHI-1* in brown cotton resulted in three fiber phenotypes of transgenic plants: brown, almost white, and green (Liu et al., 2018). In transgenic lines with almost white fibers, the *GhCHI-1* and *GhCHI-2* were significantly suppressed, whereas in transgenic lines with brown and green fibers were not. Interestingly, an anthocyanidin 3-O-glucosyltransferase (*Gh3GT*) that glucosylates the 3-position of the flavonoid C-ring was significantly up-regulated in green fiber transgenic lines. Furthermore, it was confirmed that the overexpression of the *Gh3GT* or *At3GT* gene in brown cotton lines produced green fiber. The green color of fibers could be due to the increased amount of anthocyanidins caused by the upregulation of *Gh3GT* that redirected the flux toward anthocyanidins (Liu et al., 2018; Canavar and Rausher, 2021). RNAi suppression of *GhF3H* in white cotton significantly increased naringenin content and reduced the length and micronaire of mature fibers (Tan et al., 2013). The even greater negative effect of naringenin on fiber properties was observed in brown cotton with introduced RNAi *GhF3H* copy by a cross. However, the overexpression of the *GhF3H* caused no noticeable effects on fiber phenotype (Tan et al., 2013).

Three classes of proteins, including TT2 (MYB123), TT8 (bHLH42), and TTG1 (WD40 family), often referred to as MBW complex, are primary regulators of PA biosynthesis. Experimental studies showed that TT2 and TT8 activate PA pathway genes while TTG1 stabilizes the MBW complex and is essential in maintaining MBW activity genes (Baudry et al., 2004; Yu et al., 2023). Two TT2-type MYB transcription factors (*GhMYB36* and *GhMYB10*) isolated from cotton were able to activate promoters of LAR and ANR as well as recover the seed phenotype of *Arabidopsis tt2* mutant, confirming their role in PA biosynthesis (Lu et al., 2017). In cotton, genetic studies identified at least six incompletely dominant genetic loci (*Lc1* – *Lc6*) linked to brown fiber (Kohel, 1985). The *Lc1* was linked to a 1.4 Mb inversion on chromosome A07 upstream of a TT2 homologous gene (*Gh_A07G2341*), which this inversion could activate (Hinchliffe et al., 2016). A fine-mapping study determined that two QTLs in the *Lc1* locus regulate the shades of brown color in fibers. The *qBF-A07-1* (*Gh_A07G2341* - TT2) is responsible for the brown color, whereas interaction between the *qBF-A07-1* and *qBF-A07-2* (*Gh_A07G0100* - TTG1) regulates the shades of brown fiber (Wen et al., 2018). A genome-wide association study (GWAS) revealed that epistatic interaction between *qBF-A07-1* and *qBF-A07-2* negatively impacts fiber yield and quality traits (Wen et al., 2018).

## Progress on research to enhance color diversity in NCC

The first report that inadvertently shifted the carbon flow from colorless PAs into colorful anthocyanidins in brown fibers was trying to suppress *GhCHI-1* (Liu et al., 2018). The transgenic line that failed to suppress the *GhCHI* produced green-colored cotton due to the activation of *Gh3GT*, further confirmed by transgenic overexpression of *Gh3GT* and *At3GT* in cotton. Whatever activated *3GT* in brown cotton during *CHI* suppression is unclear; nonetheless, Liu et al. (2018) showed an experimental enhancement of the anthocyanidin biosynthesis in NCC.

Another study has exploited color diversity in NCC by modulating the expression of transcription factors controlling anthocyanin biosynthesis genes. The transcription factor *GhMYB113* (*Re*), which regulates anthocyanin biosynthesis, has been overexpressed in cotton under fiber-specific promoter (Wang et al., 2022). A clear purple-red color was produced in developing fibers (5-10 DPA) of transgenic lines; however, the mature fiber turned brown. The anthocyanins content was high at 5-10 DPA and then gradually decreased, whereas PA content was low at 20 DPA and gradually increased at 25 DPA (Wang et al., 2022). The expression levels of *UFGT* (*3GT*) and *ANR* were elevated in transgenic lines. Authors suggested that anthocyanins accumulated during the early fiber developmental stage converted into PAs during the maturation and dehydration process (Wang et al., 2022).

How do we prevent the conversion of anthocyanins into PAs during the late stage of fiber development? One possible approach is activating anthocyanin biosynthesis during the later stage of fiber development, and a recent study demonstrated that it is feasible. The *GhTT2-3A* (*Gh_A07G2341)* was overexpressed under the secondary cell wall (SCW) specific promoter (*FbL2A*) in cotton; PA structural genes and PA biosynthesis were activated during the SCW stage in transgenic plants that resulted in brown mature fiber with lint percentage and fiber quality comparable to white fiber control (Yan et al., 2018). This study tried to reduce the adverse effects of pigment production on fiber cell development due to the known PA subunit’s cellular toxicity and the inhibitory role of naringenin on fiber elongation (Tan et al., 2013; Dixon and Sarnala, 2020). Yan et al. (2018) achieved the goal of producing brown fiber with improved quality. They demonstrated that the SCW stage can be used for biotechnological manipulations of PAs and other flavonoids in cotton fiber.

## Enhanced flame retardancy of NCC

What is causing the flame retardancy (FR) of NCC still remains unclear. Few studies have been published on NCC fabrics’ thermal and burning behavior. The earlier work detected a higher value of limited oxygen index for brown than for white cotton fabric (Parmar and Chakraborty, 2001). The same study suggested that heavy metal ions and color compounds could be responsible for higher FR in brown cotton (Parmar and Chakraborty, 2001). Another study tested FR properties of needle-punched (NP) and hydroentangled (H-E) nonwoven fabrics produced from fibers of brown and white cotton (Hinchliffe et al., 2015). NP is a process for manufacturing nonwoven fabrics using barbed needles, whereas H-E is high-pressure water jets for interlocking fibers together. The NP fabrics showed significantly higher FR than H-E fabrics of brown cotton. The study suggested that the unknown FR component in the brown cotton fibers is water-soluble and was removed by water during the H-E process; the higher levels of phosphorous and boron in brown cotton corresponded with enhanced FR (Hinchliffe et al., 2015).

More recent studies suggested that PA biosynthesis plays a vital role in the natural FR of brown cotton (**Figure 2**). Nam et al. (2016) demonstrated by washing fibers in water and a low concentration of sodium hydroxide solution that the condensed tannins and higher sodium content in brown cotton are responsible for the FR property. Authors suggested that condensed tannins significantly enhanced the adsorption of sodium ions to the fiber, contributing to FR (Nam et al., 2016). Another study argued that condensed tannin is not the source of FR in brown cotton since enhanced FR and anthocyanin precursors appear in developing fibers well before the brown color is detectable (Hinchliffe et al., 2016). Authors suggested that enhanced FR can be developed in white cotton by activating biosynthesis of flavonoids or PA precursors but preventing PA polymerization (source of brown color); the unknown FR component is probably sequestered by PAs or PA precursors via metal-flavonoid complexes (**Figure 2**) (Hinchliffe et al., 2016). As proof of concept, the follow-up study found four recombinant inbred lines (RILs) with enhanced FR in the multi-parent advanced generation intercross (MAGIC) population (Thyssen et al., 2023). All four RILs have white cotton fibers and enhanced FR. Authors suggested that the accumulation of colorless flavonoids might contribute to FR in these RILs; however, metabolite analysis of developing fibers has yet to confirm this hypothesis (Thyssen et al., 2023). Identifying the natural FR compound from cotton could help minimize the use of synthetic FR additives in textiles.

## Utilization of NCC

NCC has been cultivated and used for thousands of years; however, with the Industrial Revolution and the development of synthetic dyes, NCC varieties became less common as white cotton became the standard. Recently, interest in NCC has resurged due to its sustainability and environmental benefits since NCC cultivation is adaptable to dry land and organic farming (Günaydin et al., 2019; Rathinamoorthy and Parthiban, 2019). Organic NCC is softer, more breathable, and less likely to cause allergies than conventional cotton.

NCC is primarily used in high-end fashion, home textiles, upholstery fabrics, etc (Günaydin et al., 2019; Rathinamoorthy and Parthiban, 2019). The unique colors of NCC, ranging from light beige to red, rust, and brown, are highly sought by designers and consumers looking for sustainable and environmentally friendly products. These cotton varieties create a wide range of products, including clothing, bedding, and towels. Using NCC in these products adds a unique and natural element that is impossible with conventionally dyed cotton. One of the challenges of using NCC in textiles is its limited availability compared to conventional cotton. While there are efforts to increase the production of NCC, it remains a niche product in the textile industry. However, as consumer demand for sustainable and environmentally friendly products grows, the use of NCC will likely continue to increase.

Some NCC varieties possess flame-retardant properties which can be used in various applications. For example, flame-retardant textiles can be used as protective clothing for firefighters, gear for soldiers, automobile interiors, matrasses, house upholsteries, and drapes. Currently, cotton-based textiles are chemically modified to introduce multifunctional groups, making them flame-retardant. The most common method is to merge N, S, P, and Si-based polymeric, non-polymeric, polymeric/non-polymeric hybrids, inorganic, and organic/inorganic hybrids with cellulose to fabricate flame-retardant cotton textiles (Islam and van de Ven, 2021). As an eco-friendlier approach, biomass tannin and phytic acid are applied to the cotton fabric surface as a flame-retardant modification (Nam et al., 2017; Yang et al., 2023). Researchers at the U.S. Department of Agriculture’s Agricultural Research Service have recently reported studies on the FR properties of brown and four white cotton lines (Hinchliffe et al., 2015, 2016; Thyssen et al., 2023). These lines reduce the need for flame-retardant chemicals embedded in consumer products. These advancements demonstrate the potential of naturally colored cotton in creating safer and more sustainable textiles.

## Concluding remarks

The biosynthesis of PAs in brown cotton has been well characterized through transgenic analysis of structural flavonoid genes. However, little is known about how PA units are modified and transported into the vacuole and how PAs are polymerized. It has been experimentally demonstrated that the SCW biosynthesis stage of fiber development is the most suitable time for biotechnological modification of the flavonoid pathway. The anthocyanin pathway can be induced in cotton fibers by overexpressing *Re* (*GhMYB113*). In future studies, it would be interesting to overexpress *Re* and anthocyanidin modifiers during the SCW stage of fiber development to see the effects on the color of fibers. The FR has been detected in brown and white cotton fibers. The FR compound, which is yet to be identified, could be an eco-friendly alternative to synthetic FR additives in textiles. The utilization of NCC remains a niche in the textile industry. However, usage of NCC will likely continue to increase as consumer demand for sustainable and environmentally friendly products grows.

## Author contributions

MN: Writing – original draft; Writing – review & editing. DH: Writing – review & editing. GT: Writing – review & editing.
